# Inhibition of AQP1 Hampers Osteosarcoma and Hepatocellular Carcinoma Progression Mediated by Bone Marrow-Derived Mesenchymal Stem Cells

**DOI:** 10.3390/ijms17071102

**Published:** 2016-07-11

**Authors:** Alessandra Pelagalli, Anna Nardelli, Raffaela Fontanella, Antonella Zannetti

**Affiliations:** 1 Dipartimento di Scienze Biomediche Avanzate, Università degli Studi di Napoli “Federico II”, Via Pansini No. 5, 80131 Napoli, Italy; 2 Istituto di Biostrutture e Bioimmagini-CNR, Via De Amicis No. 95, 80145 Napoli, Italy; anna.nardelli@ibb.cnr.it (A.N.); raffaela.fontanella@alice.it (R.F.)

**Keywords:** BM-MSCs, osteosarcoma, hepatocellular carcinoma, AQP1, migration, invasion

## Abstract

The complex cross-talk between tumor cells and their surrounding stromal environment plays a key role in the pathogenesis of cancer. Among several cell types that constitute the tumor stroma, bone marrow-derived mesenchymal stem cells (BM-MSCs) selectively migrate toward the tumor microenvironment and contribute to the active formation of tumor-associated stroma. Therefore, here we elucidate the involvement of BM-MSCs to promote osteosarcoma (OS) and hepatocellular carcinoma (HCC) cells migration and invasion and deepening the role of specific pathways. We analyzed the function of aquaporin 1 (AQP1), a water channel known to promote metastasis and neoangiogenes. AQP1 protein levels were analyzed in OS (U2OS) and HCC (SNU-398) cells exposed to conditioned medium from BM-MSCs. Tumor cell migration and invasion in response to BM-MSC conditioned medium were evaluated through a wound healing assay and Boyden chamber, respectively. The results showed that the AQP1 level was increased in both tumor cell lines after treatment with BM-MSC conditioned medium. Moreover, BM-MSCs-mediated tumor cell migration and invasion were hampered after treatment with AQP1 inhibitor. These data suggest that the recruitment of human BM-MSCs into the tumor microenvironment might cause OS and HCC cell migration and invasion through involvement of AQP1.

## 1. Introduction

**Figure 1:**
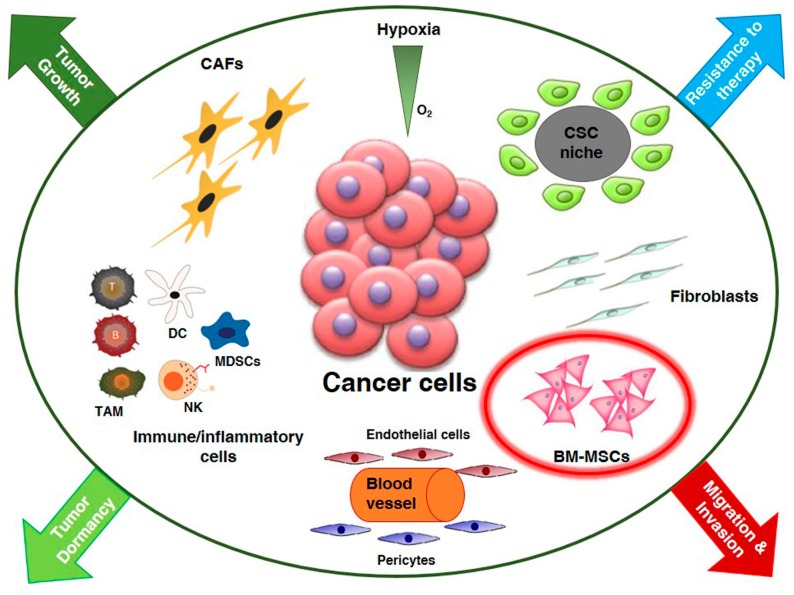
The primary tumor microenvironment. Cancer cells in primary tumor are surrounded by a complex microenvironment including multiple stromal cell types that converge to promote tumor growth and dormancy, invasion and metastasis development and resistance to therapy. Key role of bone-marrow derived mesenchymal stem cells (BM-MSCs) in tumor cell migration and invasion. Cancer stem cell (CSC); carcinoma associated fibroblasts (CAFs); dendritic cell (DC); myeloid-derived-suppressor cells (MDSCs); natural killer (NK); tumor associated macrophages (TAM).

Tumors are essentially composed of two interdependent compartments, namely tumor cells and surrounding stroma. Tumor stroma is largely a product of the host and contains connective tissue, blood vessels and several types of resident and infiltrating host cells. The most apparent function of tumor stroma is to provide a structural support to tumor cells but it has become increasingly evident that it also interacts with tumor cells and evolves with them [1]. In fact, the architecture surrounding the tumor is not static but is subjected to a continuous remodelling in response to the dynamic interplay between tumor cells and stroma [2]. While tumor cells have a clonal origin and grow accumulating mutations in different genes, stromal cells are non malignant, polyclonal and have variable origin. In tumor microenvironment (TME) there are many stromal cells including: fibroblasts, endothelial cells of the blood and lymphatic circulation, pericytes and a variety of bone marrow-derived cells that comprise macrophages, neutrophils, mast cells, myeloid-derived suppressor cells and mesenchymal stem cells (Figure 1). Specific homing to TME of bone marrow mesenchymal stem cells (BM-MSCs) and their immunosuppressive effect renders them appropriate vehicles to deliver drugs to TME [3]. Conversely, it has been demonstrated that BM-MSCs contribute to the development of cancer stem cell (CSC) niche in breast [4] and prostate carcinomas [5]. 

There is no doubt that bidirectional communication between cancer cells and surrounding stroma can promote the transfer of information for as the production of factors (growth factors, cytokines and chemokines) acting through a paracrine mechanism to modify the microenvironment. Moreover, it has demonstrated that tumor cells secrete extracelullar vesicles containing different biomolecules (proteins, RNA, DNA and lipids) [6] that may be involved in promoting cancer progression and may represent targets for therapeutic intervention [7]. Such modifications in the microenvironment can stimulate tumor initiation, proliferation and metastasis. In the last decades, many studies focused attention on the possible involvement of aquaporins (AQPs), a family of proteins characterized for their particular ability to regulate water and solute trafficking along membrane, with tumor progression [8]. Recent findings demonstrated that water homeostasis regulated by aquaporins represents an important mechanism at the basis of numerous processes such as cancer cell differentiation [9,10] and proliferation as well as apoptosis [11]. Moreover, aquaporins seem to be involved in tumor cell migration [12] and thus, in tumor spread and metastasis because of their sensitivity to changes in extracellular osmolality [13]. In particular, many studies showed a key role of AQP1 in metastatic process and neo-angiogenesis in a tumor animal model [14]. Nude mice that received AQP1-expressing melanoma cells via the tail vein developed more lung metastases at 3 weeks with respect to control mice [15]. AQP1 is strongly expressed in several human breast carcinomas, glioblastoma multiformes and several types of primary lung tumors. Moreover, it has been well demonstrated that tumors expressing AQP1 were more infiltrative compared with AQP1-null tumors [12]. Recent studies reported that AQPs might play an important role in human hepatocarcinoma cells (HCCs) [16]. Furthermore, Wu et al. (2015) [17] found that AQP1 mRNA was elevated in osteosarcoma tissue and a high level of AQP1 was associated with poor prognosis in osteosarcoma. In this study, we analyzed the involvement of AQP1 in osteosarcoma (OS) and hepatocellular carcinoma (HCC) progression due to BM-MSCs.

## 2. Results

### 2.1. Enhancement of Aquaporin 1 (AQP1) Levels in Osteosarcoma (OS) and Hepatocellular Carcinoma (HCC) Cells by Conditioned Medium from Bone Marrow-Derived Mesenchymal Stem Cells (BM-MSCs)

**Figure 2:**
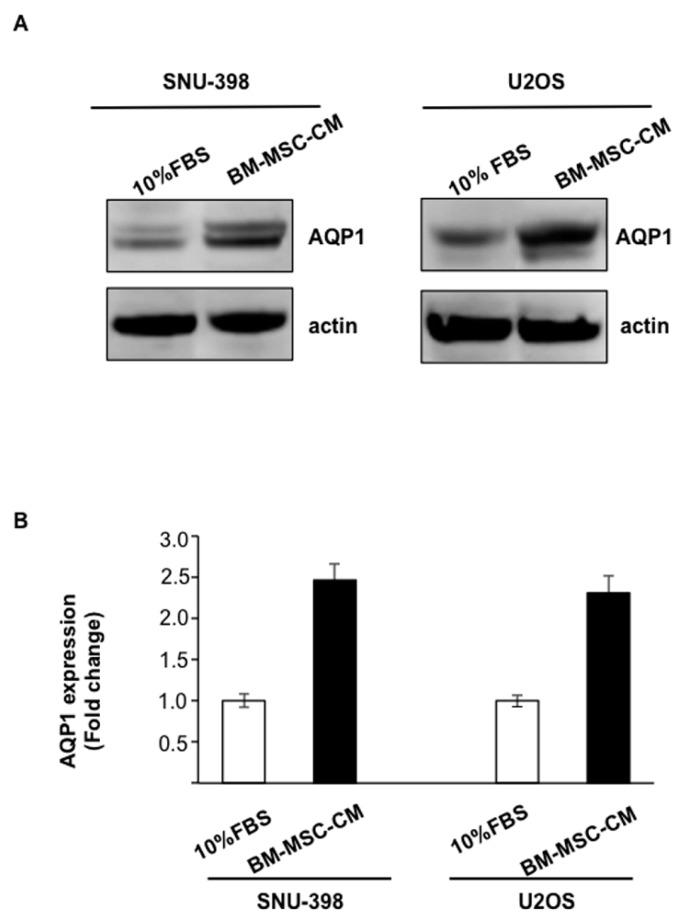
Conditioned medium (CM) from bone-marrow-derived mesenchymal stem cells (BM-MSC) increases aquaporin 1 (AQP1) levels in SNU-398 and osteosarcoma cells (U2OS) tumor cell lines. (**A**) Western blot analysis of AQP1 expression in U2OS and SNU-398 after treatment with BM-MSC-CM for 24 h. The equal protein loading was evaluated by using actin. Representative images from three independent studies; (**B**) AQP1 levels were represented as fold change respect to 10% fetal bovine serum (FBS), which was arbitrarily determined to be 1. Results are expressed as means ± SD.

We evaluated whether conditioned medium from BM-MSCs (BM-MSC-CM) could affect the expression of the water channel AQP1 in osteosarcoma cells (U2OS) and hepatocellular carcinoma cells (SNU-398). BM-MSC-CM treatment of OS and HCC cells for 24 h caused a significant increase in AQP1 protein levels (Figure 2A). We found that BM-MSCs induced a 25- and 2.3-fold enhancement in AQP1 levels in U2OS and SNU-398 cells, respectively, with respect to the control (10% fetal bovine serum (FBS)) (Figure 2B).

### 2.2. Inhibition of AQP1 Hampers OS and HCC Cell Migration Bone Marrow-Derived Mesenchymal Stem Cells-Conditioned Medium (BM-MSC-CM)-Mediated

**Figure 3:**
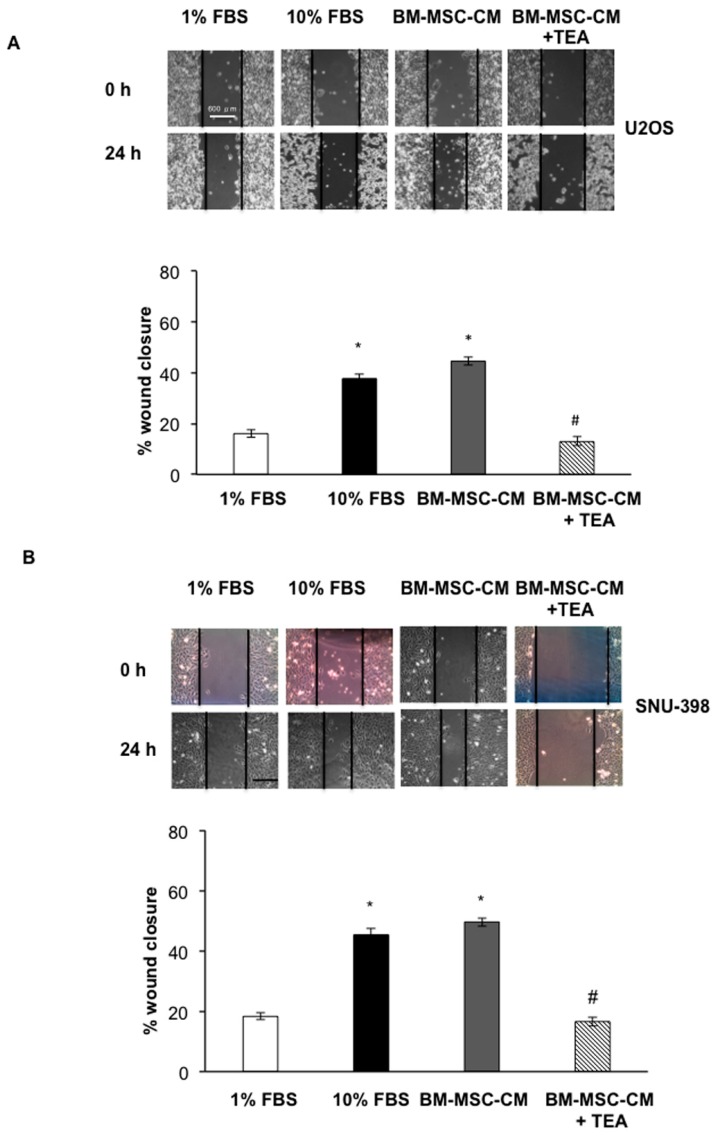
AQP1 inhibitor hampered BM-MSC-CM-mediated wound closure in U2OS and SNU-398 cells. (**A**,**B**) Scratch wounds were performed when both tumor cells were confluent and images of wounds area were captured at T = 0 and 24 h using a digital camera attached to phase contrast microscopy. The experiments were performed in the presence or absence of 100 µM tetraethylammnium chloride (TEA) used as the AQP1 inhibitor. Wound closure was measured using Image J (National Institutes of Health, Bethesda, MD, USA) (%) = 1 − (wound width tx/wound width t0) × 100. Results are expressed as means ± SD. * *p* < 0.001 and # *p* < 0.001. Scale bar = 600 µm.

Because tumor cells with high levels of AQP1 had a stronger capacity for cell migration, invasion, and metastasis [15], the BM-MSC-CM effect on wound healing was investigated. After wounding, images of U2OS and SNU-398 migration were acquired at 0 and 24 h (Figure 3A,B). The treatment with BM-MSC-CM induced a significant wound closure enhancement in U2OS cells (49.6%) with respect to control cells (1% FBS) (*p* < 0.01). Treatment with 100 µM of the AQP1 inhibitor [18] tetraethylammonium chloride (TEA) in U2OS cells cultured in the presence of BM-MSC-CM caused a significant delay in wound closure (16.6%) with respect to cells grown in the presence of BM-MSC-CM alone (*p* < 0.001) (Figure 3A). Similar to that shown in U2OS cells, BM-MSC-CM induced significant wound closure enhancement (44.5%) in SNU-398 cells. Similar to OS cells, TEA induced a significant reduction of BM-MSC-CM-mediated wound closure (13%) in HCC cells (*p* < 0.001) (Figure 3B).

### 2.3. Inhibition of AQP1 Hampers BM-MSCs-CM-Dependent OS and HCC Cell Invasion

**Figure 4:**
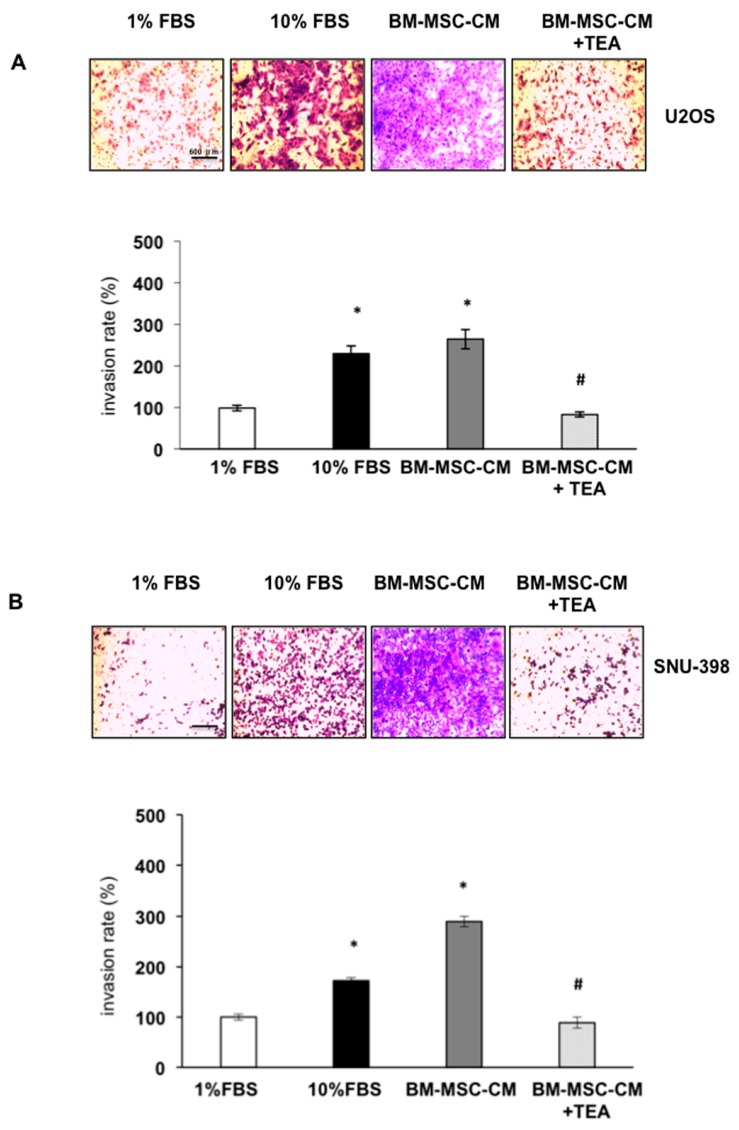
AQP1 inhibitor prevented BM-MSC-CM-mediated invasion of U2OS and SNU-398 cells. (**A**,**B**) Invasion assay was performed using a Boyden chamber pre-coated with matrigel. Tumor cells were seeded in the upper chamber whereas 1% FBS (negative control), 10% FBS (positive control) and BM-MSC-CM were added to the lower chamber as chemoattractants. Tumor cells were treated or not treated with AQP1 inhibitor TEA (100 µM). Results are expressed as means ± SD. * *p* < 0.001 and # *p* < 0.001. Scale bar = 600 µm.

To assess the BM-MSC effect on U2OS and SNU-398 cell invasiveness, a Boyden chamber with membrane pre-coated with matrigel was used. A significant increase in OS and HCC cell invasiveness with respect to the control (1% FBS supplemented medium) was observed when both tumor cell lines were seeded in the upper chamber and exposed for 48 h to BM-MSC-CM as chemoattractant (lower chamber). The AQP1 inhibitor (TEA) caused a significant decrease in the BM-MSC-mediated U2OS and SNU-398 cell invasion rate (*p* < 0.001) (Figure 4A,B).

## 3. Discussion

As in normal wound healing, tumora also activate the recruitment of host cells into the tumor microenvironment to regulate survival and proliferation [19]. Recently, bone marrow mesenchymal stem cells were shown to be recruited into primary tumor sites and contribute to the development of niches for cancer stem cells and the epithelial-to-mesenchymal transition (EMT) program [20]. In particular, growing evidence supports the hypothesis that BM-MSCs participate in TME differentiation into carcinoma-associated fibroblasts (CAFs) [21,22]. Many studies reported that BM-MSCs promote tumor proliferation and invasion of different carcinomas including breast [4,23], prostate [20,22], osteosarcoma [24,25,26] and colon [27,28,29]. Recent studies have also clarified the role of tumor cells as cells capable of modifying their environment by releasing important biomolecules from extracellular vesicles [7]. Furthermore, proteins, RNA and lipids released by tumor cells may be clinically relevant to cancer progression and useful in diagnosis of cancer and assessment of prognosis. Previous work by our group demonstrated that BM-MSCs induce OS and HCC progression through CXCR4 activation [30]. Furthermore, we observed an inhibition of the EMT program when OS and HCC cells were treated with a novel CXCR4 antagonist. It has been observed that through enhanced water flux mediated by the AQP channels, tumor cells are believed to acquire an enhanced migratory and invasive phenotype [12]. AQP1 is up-regulated in different carcinomas including: colon, breast, lung and glioblastoma multiforme [15]. Several reports found that AQP1 overexpression in tumor cells may increase tumor invasiveness and angiogenesis [31].

In the present study, we used tetraethylammonium (TEA) to block AQP1. The inhibitor effect of this molecule on AQP1 function is controversial. Brooks et al. [18], for the first time, reported that TEA reduced the water permeability of human AQP1 expressed in *Xenopus* oocytes. These data were subsequently confirmed and a much stronger AQP1 inhibition by TEA was observed [32]. Moreover, a combination of three different theoretical approaches identified the binding site for TEA in human AQP1, thus suggesting this compound as a putative lead for AQP1 selective blockers [33]. Conversely, other studies did not support the action of this molecule to inhibit AQP1 activity [34,35]. The different behavior shown by TEA to block AQP1 could be dependent on the assay system used and the cell line tested [36].

In addition, Wu et al. [17] reported that knockdown of AQP1 by siRNA in osteosarcoma cells; U2OS or MG63 cells inhibited cell proliferation and significantly inhibited cell adhesion and invasion. Furthermore, the AQP1 inhibitor AqB013 abrogated migration and invasiveness of colon cancer cells and prevented endothelial tube formation in vitro [37]. In the present study, we demonstrate that BM-MSCs-CM stimulated migration and invasion of osteosarcoma and hepatocellular carcinoma cells and their effects were hampered by the addition of the AQP1 inhibitor, thus confirming the involvement of this channel protein in the first step of the metastatic process.

## 4. Materials and Methods

### 4.1. Cell Lines and Culture Conditions 

The human osteosarcoma cells (U2OS) from Sigma-Aldrich (St. Louis, MO, USA) were grown in McCoy’s 5A. Hepatocellular carcinoma cells (SNU-398) from the American Type Culture Collection (Rockville, MD, USA), were cultured in DMEM. FBS (10%), penicillin (100 U/mL) and streptomycin (100 µg/mL) were added to both media. All cell lines were cultured in an incubator (37 °C; CO_2_ 5%).

### 4.2. Bone Marrow Mesenchymal Stem Cell Isolation and Characterization (BM-MSCs)

BM-MSCs kindly provided by the Rizzoli Orthopaedic Institute were cultured in α-modification of minimal essential medium (α-MEM, Gibco, BRL, Gaithersburg, MD, USA) supplemented with 20% FCS for 2–3 passages [38]. Moreover, flow cytofluorimetric analysis was used to evaluate stemness in BM-MSCs markers (positive markers: CD44, CD73, CD90, CD105, CD146) (negative markers: CD34 and CD45).

In order to obtain conditioned medium (CM), BM-MSCs were seeded and grown in medium with 20% FCS until 70% cell confluence and then it was replaced with FCS-free medium for 24 h.

### 4.3. Western Blot Analysis

Cell lysates were lysed in 40 mM Hepes pH 7.5, 120 mM NaCl, 5 mM MgCl_2_, 1 mM EGTA, 0.5 mM EDTA, 1% Triton X-100 containing protease (Complete Tablets-EDTA free, Roche, Basel, Switzerland) and phosphatase inhibitors (20 mM a-glycerol-3-phosphate, 2.5 mM Na-pyrophosphate) according to the method previously described [39]. After homogenization and centrifugation for 15 min at 13,000× *g* at 4 °C the protein suspension was collected and the protein content was determined using a BCA protein assay kit (Thermo, Waltham, MA, USA). Equal proteins samples (50 µg) were loaded and separated by SDS-PAGE under reducing conditions. The next step was to transfer the separated proteins to PVDF membranes. After blocking in 5% milk, primary antibodies were added. After being washed in TTBS buffer, the membranes were incubated with horseradish peroxidase-labeled antimouse or antirabbit Ig antibodies for 1 h and then protein detection was performed by ECL system (Menlo Park, CA, USA). Each band of western blot was quantified by densitometric analysis using Image J. Anti-AQP1 (sc-20810, Santa Cruz, CA, USA) and anti-β-actin (A4700, Sigma-Aldrich) were used in the experiment.

### 4.4. Wound Healing Assay

U2OS or SNU-398 cells were plated in 6-well plates and grown until confluence. Then, a linear wound was gently created in the monolayer using a sterile yellow pipette tip. After rinsing with PBS to remove the debris, the cells were incubated with culture medium containing 1% FBS, 10% FBS, BM-MSCs-CM, or TEA (100 µM) at 37 °C and 5% CO_2_ for 24 h. Images of wounds area were obtained using phase contrast microscopy. As the cell migrated to fill the scratched area, images were captured by a digital camera attached to microscope. The wound closure was quantitated in each sample as the area covered by the cells in 24 h using TScratch analysis software [40].

### 4.5. Cell Invasion Assay

24-well trans-well plates (with 8 µm pores membranes) (Corning Inc., Corning, NY, USA) were used. Membranes were pre-coated with diluted Matrigel (1:3 in PBS); U2OS and SNU-398 cells (2.5 × 10^5^) in serum-free medium were then plated in the upper chamber. In the lower chamber, the following were added as chemoattractants: medium containing 1% FBS, 10% FBS and conditioned medium obtained from BM-MSC. After 48 h incubation, the cells that had invaded the lower chamber were stained with 0.1% crystal violet, then colorant was eluted and the absorbance read at 570 nm. To block AQP1, U2OS and SNU-398 cells cells were treated with 100 µM of TEA, a specific AQP1 inhibitor. 

### 4.6. Statistics

All experiments were independently performed at least three times and the data values are presented as means ± standard deviation (SD). Statistical analysis was performed by Student’s *t* test using GraphPad Instat (La Jolla, CA, USA). *p* ≤ 0.05 was considered statistically significant.

## 5. Conclusions

We found that AQP1 levels in osteosarcoma (OS) and hepatocellular carcinoma (HCC) cells were increased when tumor cells interacted with BM-MSCs in the microenvironment. In addition, we elucidated the key role of AQP1 in BM-MSC-induced migration and invasion of OS and HCC cells. In conclusion, our study may provide a potential therapeutic strategy targeting AQP1 to hamper cross-talk between BM-MSCs and tumor cells, thus preventing the establishment of distant metastasis.

## Abbreviations

AQPs	aquaporins
α-MEM	α-modification of minimal essential medium
BM-MSCs	bone marrow-derived mesenchymal stem cells
CAFs	carcinoma associated fibroblasts
CM	conditioned medium
CSCs	cancer stem cells
DC	dendritic cell
DMEM	Dulbecco’s modified Eagle medium
ECL	enhanced chemiluminiscence
EMT	epithelial-to-mesenchymal transition
FBS	fetal bovine serum
FCS	fetal calf serum
HCC	hepatocellular carcinoma
MDSCs	myeloid-derived-suppressor cells
NK	natural killer
OS	osteosarcoma
PBS	phosphate buffered saline
SD	standard deviation
TAM	tumor associated macrophages
TEA	tetraethylammonium chloride
TME	tumor microenvironment
